# GC–MS-Based Metabonomic Profiling Displayed Differing Effects of Borna Disease Virus Natural Strain Hu-H1 and Laboratory Strain V Infection in Rat Cortical Neurons

**DOI:** 10.3390/ijms160819347

**Published:** 2015-08-17

**Authors:** Siwen Liu, Liv Bode, Lujun Zhang, Peng He, Rongzhong Huang, Lin Sun, Shigang Chen, Hong Zhang, Yujie Guo, Jingjing Zhou, Yuying Fu, Dan Zhu, Peng Xie

**Affiliations:** 1 Department of Neurology, Yongchuan Hospital, Chongqing Medical University, Chongqing 402460, China; E-Mails: 18983145528@163.com (S.L.); chongqing2012bruce@gmail.com (L.Z.); 2 Institute of Neuroscience and the Collaborative Innovation Center for Brain Science, Chongqing Medical University, Chongqing 400016, China; E-Mails: Liv.Bode@web.de (L.B.); hepeng000@sina.com (P.H.); tilamisu789456123@126.com (L.S.); iconsig@sina.com (S.C.); ASDFG43215@126.com (H.Z.); g1240725344@163.com (Y.G.); duduzjj@163.com (J.Z.); yingyuf0311@163.com (Y.F.); 3 Chongqing Key Laboratory of Neurobiology, Chongqing Medical University, Chongqing 400016, China; 4 Department of Rehabilitation, the Second Affiliated Hospital, Chongqing Medical University, Chongqing 400016, China; E-Mail: rzhuang@live.com; 5 Department of Neurology, the First Affiliated Hospital, Chongqing Medical University, Chongqing 400016, China; E-Mail: zhudan25@126.com

**Keywords:** borna disease virus, neuron, metabonomic, GC-MS, rat

## Abstract

Borna disease virus (BDV) persists in the central nervous systems of a wide variety of vertebrates and causes behavioral disorders. Previous studies have revealed that metabolic perturbations are associated with BDV infection. However, the pathophysiological effects of different viral strains remain largely unknown. Rat cortical neurons infected with human strain BDV Hu-H1, laboratory BDV Strain V, and non-infected control (CON) cells were cultured *in vitro*. At day 12 post-infection, a gas chromatography coupled with mass spectrometry (GC–MS) metabonomic approach was used to differentiate the metabonomic profiles of 35 independent intracellular samples from Hu-H1-infected cells (*n* = 12), Strain V-infected cells (*n* = 12), and CON cells (*n* = 11). Partial least squares discriminant analysis (PLS-DA) was performed to demonstrate discrimination between the three groups. Further statistical testing determined which individual metabolites displayed significant differences between groups. PLS-DA demonstrated that the whole metabolic pattern enabled statistical discrimination between groups. We identified 31 differential metabolites in the Hu-H1 and CON groups (21 decreased and 10 increased in Hu-H1 relative to CON), 35 differential metabolites in the Strain V and CON groups (30 decreased and 5 increased in Strain V relative to CON), and 21 differential metabolites in the Hu-H1 and Strain V groups (8 decreased and 13 increased in Hu-H1 relative to Strain V). Comparative metabonomic profiling revealed divergent perturbations in key energy and amino acid metabolites between natural strain Hu-H1 and laboratory Strain V of BDV. The two BDV strains differentially alter metabolic pathways of rat cortical neurons *in vitro*. Their systematic classification provides a valuable template for improved BDV strain definition in future studies.

## 1. Introduction

Borna disease virus (BDV) is a neurotropic, enveloped, non-segmented, negative-stranded RNA virus that causes chronic, persistent infections of neurons and glial cells. BDV infection has been reported in a range of animal species across a broad global geographic distribution [1], including China [2,3]. Previous studies have demonstrated that BDV can infect many mammalian species [4]. Its extraordinarily broad host spectrum includes horses, sheep, cattle, cats, ostrich, macaques [5,6,7], and human [8,9]. In addition to BDV, another species within the genus *Bornavirus* of the family *Bornaviridae* has been identified, the avian bornavirus (ABV), and has for the first time been associated with proventricular dilatation disease (PDD), a fatal disorder threatening domesticated and wild psittacine birds worldwide [10]. ABVs were sharing less than 70% genetic identity with the very closely related mammalian BDVs. To our knowledge they have thus far not been subjected to metabonomic profiling. Another remarkably interesting issue is that BDV is an evolutionarily very old virus with a suggested co-evolution of more than 40 million years in primate ancestor hosts up to humans [11], according to functional Endogenous Borna-like nucleoprotein (EBLNs). The impact of EBLNs in human and animal exogenous BDV infection remained as yet unclarified, and *in vitro* metabolomics studies are lacking as well.

Infected mammalian animal hosts develop a wide spectrum of neurological disorders ranging from immune-mediated diseases to behavioral alterations without inflammation [12]. However, the mechanism(s) underlying BDV’s pathogenesis are not well understood. The virus manipulates cholinergic, GABAergic, and monoaminergic neurotransmitter pathways, as significant alterations occur in choline acetyltransferase (ChAT), acetylcholinesterase (AchE), glutamic acid decarboxylase (GAD), norepinephrine, and serotonin levels [13]. Remarkably, there is also immune-histopathological evidence in the rat model that the excitatory glutamate system in hippocampal neurons is a major target of BDV, as major proteins (N and P) are apparently binding to a particular glutamate receptor (kainate1, KA1) which is present in CA3 (Cornu Ammon3)and dentate gyrus areas but not in the CA1 area [14].

These early studies were comparable in that they were using a laboratory virus (BDV Strain V) which was the first strain completely sequenced back to 1994 [15]. Due to unique features within the order *Mononegavirales*, the International Committee on Taxonomy of Viruses (ICTV) consequently established a novel family, *Bornaviridae*, to include a single genus, *Bornavirus*, for a single species, Borna disease virus (BDV), with Strain V serving as the reference virus [16]. The current taxonomy is meanwhile subject to ongoing debate [17], given the identification of genetically divergent bornaviruses, like the aforementioned ABVs [10]. Taxonomy issues are beyond the scope of this study. However, focusing on the metabonomic profiles of two BDVs of completely different origin does require a classification nomenclature below the species level which at the same time provides the entire history of these BDVs. As this study compares a natural human strain (BDV Hu-H1) [18] and a non-natural laboratory-adapted cross-species strain of equine origin (BDV Strain V) [4,19,20], we for the first time introduce their systematic classification, analogously to what has been proposed for natural and laboratory-adapted strains of Ebola virus (EBOV) assigned to the family of *Filoviridae* [21,22].

Our previous studies have demonstrated that BDV Hu-H1 perturbs energy metabolites and amino acids in cultured human oligodendroglia (OL) cells [23]. Further evidence by proteomics-based profiling confirmed the Hu-H1-induced perturbation of host energy metabolism, and additionally found disturbed host cell proliferation, possibly through impaired nuclear translocation of pERK (protein kinase R-like ER kinase) [24]. A recent publication could demonstrate that this human BDV strain, Hu-H1, also impacts important post-translational modifications like acetylation upon infection. The acetylome of infected OL cells was manipulated towards higher energy and transporter levels [25]. Most notably, human strain BDV Hu-H1 and laboratory strain (Str. V) were found to induce opposite effects, namely decreased *vs.* increased proliferation, and increased *vs.* decreased apoptosis, respectively [26].

Metabonomics, which enables the simultaneous quantitative measurement of numerous low molecular weight molecules within diseased samples [27], have been used to analyze the changes in whole metabolic patterns in response to viral infection [28,29]. Gas chromatography–mass spectrometry (GC–MS), liquid chromatography-mass spectrometry (LC–MS), and nuclear magnetic resonance (NMR) coupled with multivariate statistical methods have been extensively applied in metabonomic research [30]. GC–MS, which has been widely applied because of its high sensitivity, peak resolution, and reproducibility compared with other methods, has become one of the most popular metabonomic techniques [31]. The human virus, BDV Hu-H1, has been employed in an earlier metabonomic approach to characterize metabolic alterations in oligodendrocytes and RD (rhabdomyosarcoma) human cell lines [23]. Furthermore, GC–MS-based profiling of metabolic changes in three brain regions of post-natally infected rats at day 56 post infection has also used this human strain and found significant perturbations in nucleotide, amino acid and lipid metabolites [32]. A GC–MS approach was also applied to analyze metabolic changes in the hippocampus of naturally infected asymptomatic horses, revealing differential metabolites mainly involved in glutamate and lipid metabolism [33]. However, no reports comparing different BDV strains with regard to neuronal metabolic changes have been published in BDV research so far.

Therefore, in this study, metabolites were profiled in rat cortical neurons infected with a natural (Hu-H1) and a non-natural strain of BDV (Strain V), respectively. We applied a GC–MS metabonomic method coupled with principal component analysis (PCA) and orthogonal partial least-squares discriminant analysis (OPLS-DA) as well as statistical analysis, and analyzed whether and how either strain alters metabolic pathways in neurons. The different origin and history of these strains should provide valuable information for future studies on BDV differences below the species level.

## 2. Results

### 2.1. Taxonomical Classification of Borna Disease Virus (BDV) Strains Hu-H1 and Strain V

The major focus of the study is to analyze whether the metabonomic profile of BDV-infected neurons differs between viruses. Therefore, analyzing the history of these viruses as well as their taxonomical classification below the species level was considered to be of utmost importance and became part of the study. BDV Hu-H1 had been recovered in 1994 from freshly isolated white blood cells of a female bipolar I disorder patient in Berlin, Germany, who was admitted to hospital during a severe depressive episode. Isolation of soundly replicating human virus (Hu-H1) had been achieved by co-cultivation techniques using OL cells after at least 10–12 blind passages [18]. Hu-H1 had been recovered from one of a set of six PBMC (peripheral blood mononuclear cell) samples within a narrow time span, three of which were testing positive for BDV RNA and protein [18]. Original RNAs as well as the RNAs of the isolate passages (p11–p25) had been found to display identical nucleotide sequences [34]. At the molecular level, Hu-H1 had shown few meaningful point mutations compared to laboratory Strain V and a second lab strain (He/80) [34], and at the host level, Hu-H1 (p25) had shown differing pathogenicity, namely inducing behavioral changes in rabbits but no deadly disease as compared to Str. V [18]. Lab Str. V originated from the brain of a horse with fatal Borna disease in Giessen, Germany in 1927 and had been primarily adapted to the rabbit through multiple *in vivo* passaging using brain suspension of intracerebrally infected 3–6-week-old rabbits [19]. The strain had been further adapted to the rat through six passages using brain suspension of intracerebrally infected suckling Wistar rats [20], later followed by *in vitro* passaging (p10–p25) in a human oligodendroglial (OL) cell line. This virus was completely sequenced in 1994 [15]. According to different biological properties following general definition [35], different host origin and source, natural and laboratory history, BDV Hu-H1 and Str. V could be considered authentic virus strains, regardless of a low genetic difference below 5%. Following these taxonomic principles of a standardized nomenclature, we for the first time introduce a systematic classification of natural BDV Hu-H1 and non-natural BDV Strain V, as proposed for Ebola virus (EBOV) natural and laboratory strains below the species level [21,22]. In contrast to Kuhn *et al.* 2013 [21,22], the virus source is also indicated, as it could be either blood (PBMC) or brain. The medium-length designated name for Hu-H1 is referring to virus identity, isolation host, sampling location, sampling year, genetic variant, isolate number and source. For Strain V, the name additionally refers to the laboratory animals used to create the laboratory-adapted variant. Details are given in Table 1. The passages of either virus used in this study are further described in the “Experimental Section”.

**Table 1:** Taxonomy and history of Borna disease virus (BDV) strains Hu-H1 and Strain V.

Template	Hu-H1 (Abbreviation)	Strain V (Abbreviation)
Order	Mononegavirales	Mononegavirales
Family	*Bornaviridae*	*Bornaviridae*
Genus	*Bornavirus*	*Bornavirus*
Species	Borna disease virus (BDV)	BDV
Virus	BDV	BDV
Strain	BDV Hu-H1 (natural)	BDV Strain V (non-natural)
Isolation host	*Homo sapiens* (H. sapiens)	*Equus ferus* (E. ferus)
Isolation source	PBMCs	Brain
Sampling location	Germany (DEU), Berlin	Germany (DEU), Giessen
Sampling year	1994	1927
Genetic variant	Patient H1, female, 45 years., depressive episode, bipolar disorder (DSM IV 296.64), Berlin 1994, 3660-PBMC, passaged in human oligodendroglial (OL-221) cells (virus p11–p25), (stable virus titer from p11 on)	Horse V, fatal Borna disease (encephalomyelitis) (-hist), Giessen horse V brain, passaged in rabbits (brain, (20–50)×), rats (brain 6×), OL-221 cells (virus p10–p25)
Suffix	Passaged in OL cells (-tc)	Passaged in laboratory animal hosts and OL cells (-lab)
Pathology in rabbits	Behavioral disease (3 weeks p.i.), Hu-H1/OL p25 used	Fatal disease (3 weeks p.i.), Strain V/OL p12 used
GenBank accession No.	U58594, L76234, L76228, L76237	U04608 (full-length)
Full designated name	Borna disease virus Hu-H1/H. sapiens-tc/DEU/1994/Berlin-Hu-H1-94 3660-PBMC	Borna disease virus Strain V/E. ferus VECTOR/O. cuniculus-lab/Wistar rat-lab/DEU/1927/Giessen-horse V-brain
Medium-length designation	BDV Hu-H1/Hsap-tc/DEU/94/Ber-Hu-H1-94 3660-PBMC	BDV Str. V/VECTOR/O. cun-lab/Wistar-lab/DEU/27/Gie-horse V-brain
Passage history of virus stock provided	OL-221/Hu-H1-94 3660, passage p75, 1994, provided 2010 to CQMU	OL-221/Strain V, passage p113, 1998, provided 2010 to CQMU
Passage grown at CQMU and used as virus source	OL-221/Hu-H1-94 3660, passage 77 in OL-221 passage 112	OL-221/Strain V, passage 115 in OL-221 passage 116

BDV: Borna disease virus; PBMC: peripheral blood mononuclear cell; DEU: Germany; DSM: diagnostic and statistical manual of mental disorders; p.i.: post-infection; CQMU: Chongqing Medical University.

### 2.2. Immunofluorescence Assay

**Figure 1:**
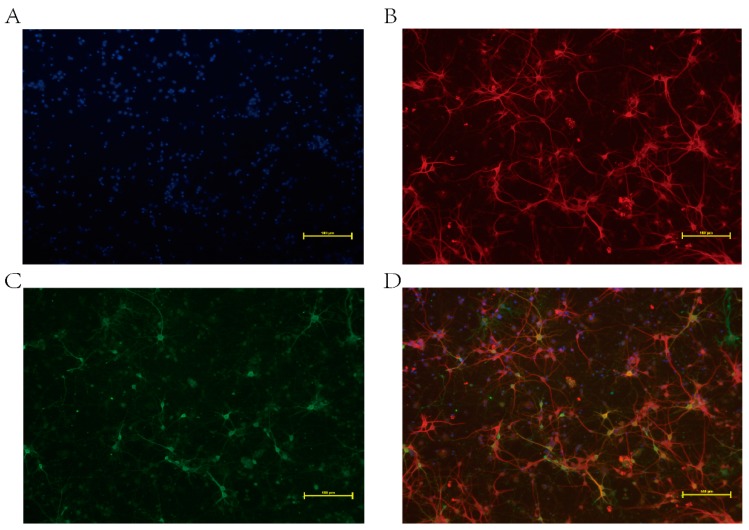
Immunofluorescence analysis of Borna disease virus (BDV)-infected neurons on day 12 post-infection. (**A**) Nuclei stained with DAPI (4′,6-diamidino-2-phenylindole) (blue); (**B**) Neurons marked with chicken polyclonal MAP-2 (neuron-specific marker) followed by a FITC (Fluorescein isothiocyanate isomer I)-labeled secondary antibody (red, goat anti-chicken); (**C**) BDV P40 detected with a primary monoclonal antibody followed by a FITC-labeled secondary antibody (green, goat anti-mouse); (**D**) Merged image. Scale bars: 100 μm.

Our previous study showed that BDV infection was first detectable on day 6 in BDV P40-positive neurons composing less than 4%. Between days 6 and 9, BDV spread rapidly, and by day 12, almost 100% of the cells were infected [36]. The immunofluorescence assay was then applied at day 12 post-infection. The percentage of neurons was determined through observation of randomly-selected cells across three independent experiments. The results showed the purity of neurons was more than 90%, and all neurons were infected with BDV (Figure 1).

### 2.3. Metabonomic Analysis

**Figure 2:**
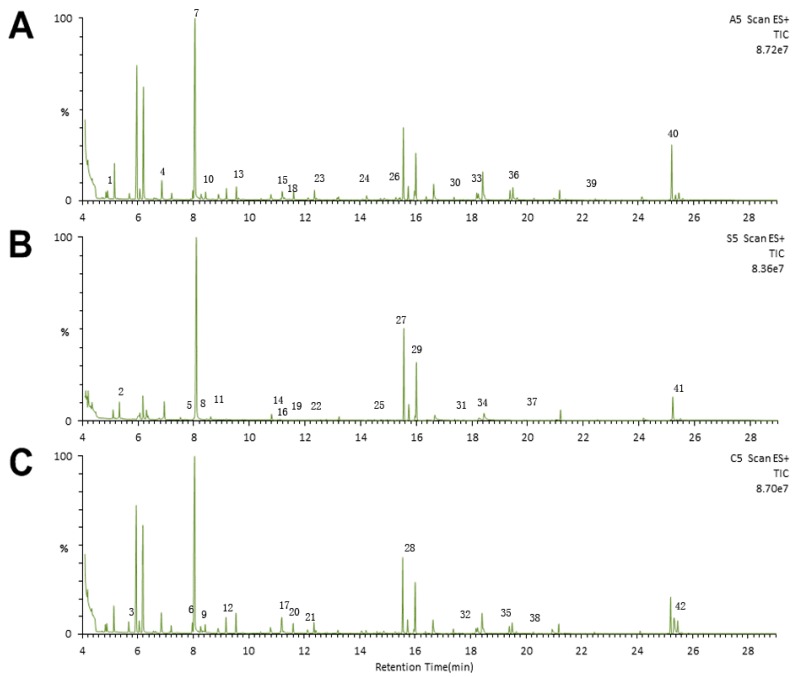
Representative gas chromatography–mass spectrometry (GC–MS) total ion chromatograms. Chromatograms from the (**A**) Hu-H1 group; (**B**) Strain V group; and (**C**) non-infected control (CON) group. (**1**) Pyruvic acid; (**2**) Glycolic acid; (**3**) l-Alanine; (**4**) l-Valine; (**5**) Ethanolamine; (**6**) l-Leucine; (**7**) Glycerol; (**8**) l-Isoleucine; (**9**) l-Proline; (**10**) Glycine; (**11**) Methylsuccinic acid; (**12**) Serine; (**13**) l-Threonine; (**14**) Malic acid; (**15**) l-Methionine; (**16**) Pyroglutamic acid; (**17**) l-Aspartic acid; (**18**) γ-Aminobutyric acid; (**19**) Creatinine; (**20**) l-Cysteine; (**21**) 3-Hydroxy-3-methylglutaric acid; (**22**) l-Glutamic acid; (**23**) Phenylalanine; (**24**) Glycerol-3-phosphate; (**25**) Citric acid; (**26**) d-Fructose; (**27**) d-Glucose; (**28**) l-Lysine; (**29**) Sorbitol; (**30**) Myo-Inositol; (**31**) Margaric acid; (**32**) Ribulose-5-phosphate; (**33**) Oleic acid; (**34**) Octadecanoic acid; (**35**) d-Fructose-6-phosphate; (**36**) d-Glucose-6-phosphate; (**37**) Eicosanoic acid; (**38**) Myo-Inositol-1-phosphate; (**39**) d-Lactose; (**40**) Cholesterol; (**41**) Lanosterol; (**42**) Desmosterol.

**Figure 3:**
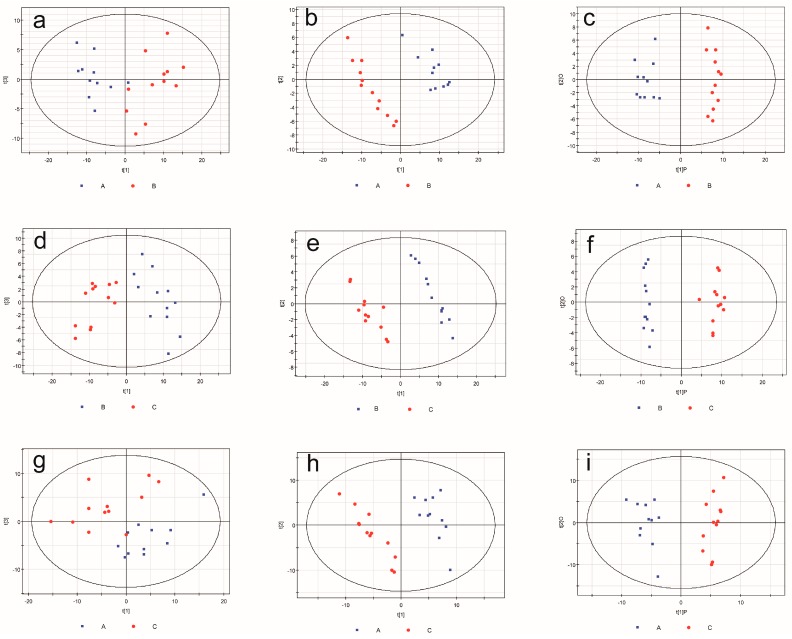
Multivariate statistical analysis. Principal component analysis (PCA) scores plot derived from GC–MS spectra of rat cortical neurons infected from (**a**) Hu-H1 and Strain V; (**d**) Strain V and non-infected control (CON); and (**g**) Hu-H1 and CON. Paired partial least-squares discriminant analysis (PLS-DA) model showing a clear separation between rat cortical neurons infected from (**b**) Hu-H1 and Strain V; (**e**) Strain V and CON; and (**h**) Hu-H1 and CON. Orthogonal partial least-squares discriminant analysis (OPLS-DA) model showing clear separation between rat cortical neurons infected with (**c**) Hu-H1 and Strain V; (**f**) Strain V and CON; and (**i**) Hu-H1 and CON. (A represents Hu-H1; B represents Strain V; C represents non-infected control (CON).)

Visual inspections of total ion current (TIC) chromatograms from both groups are provided. All displayed strong signals for analysis as well as large peak capacity and good reproducibility in retention time (Figure 2). After excluding internal standards, 220 individual peaks were detected, which existed in over 80% of the samples in each group. These peaks were used in the subsequent multivariate analysis. Clear differences were presented by the PCA score plots in the BDV Hu-H1 and Strain V groups (R2X = 0.638; Figure 3a), Strain V and CON groups (R2X = 0.622; Figure 3d), and Hu-H1 and non-infected control (CON) groups (R2X = 0.613; Figure 3g). The pair-wise PLS-DA score plots revealed statistical differences between the BDV Hu-H1 and Strain V groups (R2Y = 0.946, Q2 = 0.821; Figure 3b), Strain V and CON groups (R2Y = 0.982, Q2 = 0.926; Figure 3e), and Hu-H1 and CON groups (R2Y = 0.995, Q2 = 0.936; Figure 3h). OPLS-DA analysis indicated that this model was efficient and showed clear separation between the BDV Hu-H1 and Strain V groups (R2Y = 0.945, Q2 = 0.791; Figure 3c), Strain V and CON groups (R2Y = 0.981, Q2 = 0.934; Figure 3f), and Hu-H1 and CON groups (R2Y = 0.965, Q2 = 0.867; Figure 3i). (R2Y is the cumulative model variation in Y, and Q2 is the cumulative predicted variation. The values of these parameters approaching parameters approaching 1.0 indicate a stable model with predictive reliability.)

According to the OPLS-DA analysis, significant differential metabolites were identified. Table 2 shows the differences between either virus and the control. Thirty-one differential metabolites in the BDV Hu-H1 and CON groups (21 decreased and 10 increased in BDV Hu-H1 relative to CON) were identified, and 35 differential metabolites in the Strain V and CON groups (30 decreased and 5 increased in Strain V relative to CON). For a better overview, Table 3 summarizes the key commonly altered metabolites of Hu-H1 and Strain V compared to CON. Finally, Table 4 shows the differences between the two virus strains. We identified 21 metabolites which differed between the Hu-H1 and Strain V groups (8 decreased and 13 increased in Hu-H1 relative to Strain V).

**Table 2:** Key differential metabolites detected by gas chromatography–mass spectrometry (GC–MS) for Hu-H1/Con and Strain V/Con.

**Hu-H1/Con**	**Metabolite**	**RT (min)**	**MZ**	**VIP-Value**	***p*-Value (*t*-test)**	Fold Change *
				**(OPLS-DA)**		
1	Citric acid	14.63	273	2.42	1.1 × 10^−10^	−1.49
2	l-CysteineL	11.61	218	1.86	0.000129	−1.34
3	Pyruvic acid	5.05	174	2.19	4.58 × 10^−^^7^	−1.29
4	l-Aspartic acid	11.2	233	2.14	0.00000144	−1.18
5	Desmosterol	25.47	343	1.94	0.0000461	−1.16
6	Margaric acid	17.6	117	2.35	3.73 × 10^−^^9^	−1.03
7	Glycerol-3-phosphate	14.08	357	1.61	0.00167	−0.8
8	Sorbitol	15.95	319	1.23	0.022	−0.77
9	l-Alanine	5.69	116	1.95	0.0000402	−0.67
10	Myo-Inositol	17.38	305	1.87	0.000112	−0.66
11	Serine	9.18	204	2.03	0.0000118	−0.58
12	Phenylalanine	12.43	218	1.16	0.0334	−0.56
13	l-Threonine	9.54	218	1.84	0.000159	−0.53
14	Pyroglutamic acid	11.19	156	1.27	0.018	−0.49
15	Malic acid	10.82	233	1.31	1.46 × 10^−^^2^	−0.47
16	l-Methionine	11.15	176	1.23	2.31 × 10^−^^2^	−0.46
17	l-Proline	8.29	142	1.15	3.45 × 10^−^^2^	−0.31
18	Octadecanoic acid	18.41	117	1.03	3.77 × 10^−^^3^	−0.29
19	l-Valine	7.21	144	1.41	7.71 × 10^−^^3^	−0.24
20	l-Leucine	7.97	158	1.24	2.22 × 10^−^^2^	−0.21
21	l-Isoleucine	8.27	158	1.16	3.36 × 10^−^^2^	−0.2
22	Eicosanoic acid	20.1	117	1.17	3.19 × 10^−^^2^	0.27
23	d-Lactose	22.47	204	1.17	3.16 × 10^−^^2^	0.31
24	d-Glucose-6-phosphate	19.65	315	1.15	3.49 × 10^−^^2^	0.49
25	d-Fructose-6-phosphate	19.4	315	1.25	1.99 × 10^−^^2^	0.54
26	d-Fructose	15.28	103	1.4	7.96 × 10^−^^3^	0.54
27	Ethanolamine	7.9	174	1.35	1.13 × 10^−^^2^	0.77
28	3-Hydroxy-3-methylglutaric acid	12.16	247	1.86	1.23 × 10^−^^4^	0.94
29	d-Glucose	15.43	205	1.68	8.27 × 10^−^^4^	0.95
30	Lanosterol	25.35	458	1.9	7.37 × 10^−^^5^	0.96
31	Methylsuccinic acid	8.69	261	1.86	1.42 × 10^−^^4^	1.79
**Strain V/Con**	**Metabolite**	**RT (min)**	**MZ**	**VIP-Value**	***p*-Value (*t*-test)**	Fold Change *
				**(OPLS-DA)**		
1	d-Glucose-6-phosphate	19.5	387	1.61	7.05 × 10^−12^	−3.59
2	d-Fructose-6-phosphate	19.4	315	1.59	3.26 × 10^−^^11^	−3.53
3	l-Cysteine	11.61	218	1.44	1.89 × 10^−^^7^	−3.31
4	γ-Aminobutyric acid	11.27	174	1.57	2.04 × 10^−10^	−3.22
5	Citric acid	14.63	273	1.66	4.82 × 10^−^^16^	−3.22
6	l-Lysine	15.6	317	1.45	1.34 × 10^−^^7^	−2.99
7	Desmosterol	25.47	343	1.5	1.60 × 10^−^^8^	−2.84
8	Pyruvic acid	5.05	174	1.09	4.94 × 10^−^^5^	−2.4
9	Phenylalanine	12.43	218	1.38	1.82 × 10^−^^6^	−2.37
10	Creatinine	11.6	115	1.47	6.99 × 10^−^^8^	−2.27
11	l-Aspartic acid	11.2	232	1.52	1.61 × 10^−^^8^	−2.06
12	l-Methionine	11.15	176	1.42	3.76 × 10^−^^7^	−1.97
13	Serine	9.18	204	1.59	4.36 × 10^−^^11^	−1.7
14	Malic acid	10.82	233	1.38	1.44 × 10^−^^6^	−1.64
15	l-Alanine	5.69	116	1.51	1.14 × 10^−^^8^	−1.56
16	d-Fructose	15.28	103	1.6	8.56 × 10^−^^12^	−1.51
17	l-Glutamic acid	12.36	246	1.17	2.07 × 10^−^^4^	−1.51
18	l-Threonine	9.54	218	1.55	6.11 × 10^−^^10^	−1.41
19	d-Glucose	15.43	205	1.46	1.15 × 10^−^^7^	−1.4
20	Glycerol-3-phosphate	14.08	357	1.27	3.36 × 10^−^^5^	−1.28
21	l-Proline	8.29	142	1.4	9.06 × 10^−^^7^	−1.22
22	Myo-Inositol	17.38	305	1.41	6.71 × 10^−^^7^	−1.2
23	Ribulose-5-phosphate	17.72	357	1.24	5.19 × 10^−^^5^	−0.99
24	Margaric acid	17.6	117	1.42	4.04 × 10^−^^7^	−0.97
25	Myo-Inositol-1-phosphate	20.26	318	1.19	1.47 × 10^−^^4^	−0.96
26	l-Valine	7.21	144	1.51	7.91 × 10^−^^9^	−0.95
27	l-Leucine	7.97	158	1.46	9.99 × 10^−^^8^	−0.91
28	Pyroglutamic acid	11.19	156	1.15	3.22 × 10^−^^4^	−0.91
29	l-Isoleucine	8.27	158	1.45	1.34 × 10^−^^7^	−0.78
30	Oleic acid	18.2	117	1.15	3.09 × 10^−^^3^	−0.55
31	Eicosanoic acid	20.1	117	1.02	1.95 × 10^−^^3^	0.45
32	Glycerol	8.05	205	1.11	5.74 × 10^−^^4^	0.56
33	Glycolic acid	5.35	177	1.11	5.85 × 10^−^^4^	0.57
34	Ethanolamine	7.9	174	1	2.52 × 10^−^^3^	0.77
35	Methylsuccinic acid	8.69	261	1.25	5.18 × 10^−^^5^	1.64

* Fold change was calculated as the logarithm of the average mass response (area) ratio between the two classes (*i.e.*, fold change = log2(Hu-H1/Con)). Thus, positive fold-change values indicate significantly higher levels in Hu-H1 relative to Con, and negative fold change values indicate significantly lower levels in Hu-H1 relative to Con.

**Table 3:** Key commonly altered metabolites detected by GC–MS for Hu-H1 and Strain V.

Hu-H1/Strain V	Metabolite	RT (min)	MZ	VIP-Value (OPLS-DA)		*p*-Value (*t*-test) Hu-H1 Strain V		Fold Change Hu-H1/CON Strain V/CON	
				Hu-H1	Strain V				
1	Citric acid	14.63	273	2.42	1.66	1.10 × 10^−10^	4.82 × 10^−1^^6^	−1.49	−3.22
2	l-CysteineL	11.61	218	1.86	1.44	0.000129	1.89 × 10^−^^7^	−1.34	−3.31
3	Pyruvic acid	5.05	174	2.19	1.09	4.58 × 10^−^^7^	4.94 × 10^−^^5^	−1.29	−2.4
4	l-Aspartic acid	11.2	233	2.14	1.52	0.00000144	1.61 × 10^−^^8^	−1.18	−2.06
5	Desmosterol	25.47	343	1.94	1.5	0.0000461	1.60 × 10^−^^8^	−1.16	−2.84
6	Margaric acid	17.6	117	2.35	1.42	3.73 × 10^−^^9^	4.04 × 10^−^^7^	−1.03	−0.97
7	Glycerol-3-phosphate	14.08	357	1.61	1.27	0.00167	3.36 × 10^−^^5^	−0.8	−1.28
8	l-Alanine	5.69	116	1.95	1.51	0.0000402	1.14 × 10^−^^8^	−0.67	−1.56
9	Myo-Inositol	17.38	305	1.87	1.41	0.000112	6.71 × 10^−^^7^	−0.66	−1.2
10	Serine	9.18	204	2.03	1.59	0.0000118	4.36 × 10^−^^11^	−0.58	−1.7
11	Phenylalanine	12.43	218	1.16	1.38	0.0334	1.82 × 10^−^^6^	−0.56	−2.37
12	l-Threonine	9.54	218	1.84	1.55	0.000159	6.11 × 10^−10^	−0.53	−1.41
13	Pyroglutamic acid	11.19	156	1.27	1.15	0.018	3.22 × 10^−^^4^	−0.49	−0.91
14	Malic acid	10.82	233	1.31	1.38	1.46 × 10^−^^2^	1.44 × 10^−^^6^	−0.47	−1.64
15	l-Methionine	11.15	176	1.23	1.42	2.31 × 10^−^^2^	3.76 × 10^−^^7^	−0.46	−1.97
16	l-Proline	8.29	142	1.15	1.4	3.45 × 10^−^^2^	9.06 × 10^−^^7^	−0.31	−1.22
17	l-Valine	7.21	144	1.41	1.51	7.71 × 10^−^^3^	7.91 × 10^−^^9^	−0.24	−0.95
18	l-Leucine	7.97	158	1.24	1.46	2.22 × 10^−^^2^	9.99 × 10^−^^8^	−0.21	−0.91
19	l-Isoleucine	8.27	158	1.16	1.45	3.36 × 10^−^^2^	1.34 × 10^−^^7^	−0.2	−0.78
20	Eicosanoic acid	20.1	117	1.17	1.02	3.19 × 10^−^^2^	1.95 × 10^−^^3^	0.27	0.45
21	Ethanolamine	7.9	174	1.35	1	1.13 × 10^−^^2^	2.52 × 10^−^^3^	0.77	0.77
22	Methylsuccinic acid	8.69	261	1.86	1.25	1.42 × 10^−^^4^	0.0000518	1.79	1.64

**Table 4:** Key differential metabolites detected by GC–MS for Hu-H1/Strain V.

Hu-H1/Strain V	Metabolite	RT (min)	MZ	VIP-Value	*p*-Value (*t*-test)	Fold Change *
				(OPLS-DA)		
1	Glycolic acid	5.35	177	1.36	3.71 × 10^−^^5^	−0.75
2	Myo-Inositol-1-phosphate	20.26	318	1.06	8.76 × 10^−^^4^	−0.68
3	d-Fructose-6-phosphate	19.4	315	1.6	4.89 × 10^−^^5^	−0.62
4	Glycerol	8.05	205	1.11	1.86 × 10^−^^5^	−0.37
5	Cholesterol	25.21	129	1.05	2.42 × 10^−^^2^	−0.36
6	Desmosterol	25.47	343	1.6	3.96 × 10^−^^2^	−0.34
7	Lanosterol	25.35	458	1.26	7.60 × 10^−^^2^	−0.25
8	Citric acid	14.63	273	1.53	1.62 × 10^−1^	−0.2
9	d-Glucose-6-phosphate	19.5	387	1.59	7.22 × 10^−^^2^	0.39
10	Myo-Inositol	17.38	305	1.09	4.37 × 10^−^^2^	0.48
11	l-Isoleucine	8.27	158	1.41	1.43 × 10^−^^5^	0.58
12	Ribulose-5-phosphate	17.72	357	1.2	1.52 × 10^−^^3^	0.61
13	l-Leucine	7.97	158	1.41	1.25 × 10^−^^5^	0.7
14	l-Valine	7.21	144	1.53	5.61 × 10^−^^7^	0.71
15	Glycine	8.43	174	1.5	1.58 × 10^−^^6^	0.83
16	d-Fructose	15.28	103	1.55	1.08 × 10^−^^3^	0.88
17	l-Threonine	9.54	218	1.39	2.11 × 10^−^^5^	0.89
18	l-Alanine	5.69	116	1.43	8.28 × 10^−^^6^	0.89
19	l-Proline	8.29	142	1.37	3.67 × 10^−^^5^	0.92
20	Serine	9.18	204	1.49	1.66 × 10^−^^6^	1.13
21	d-Glucose	15.43	205	1.51	3.58 × 10^−^^8^	1.14

* Fold change was calculated as the logarithm of the average mass response (area) ratio between the two classes (*i.e.*, fold change = log2(Hu-H1/Con)). Thus, positive fold-change values indicate significantly higher levels in Hu-H1 relative to Con, and negative fold change values indicate significantly lower levels in Hu-H1 relative to Con.

## 3. Discussion

BDV is a remarkable, evolutionary and old [11] RNA virus which preferentially and persistently infects neurons and other brain cells of a broad variety of mammalian hosts including humans [1,4,5,6,7,8,9]. After the discovery of divergent bornaviruses in avian hosts [10], the taxonomy within the family *Bornaviridae* became the subject of debate [17]. This study’s scope is not to add to this scholarly debate even though it does deal with taxonomical issues below the species level of BDV, as two strains are compared. One is the non-natural laboratory-adapted reference Strain V [4], which is of horse origin [19] and was the first to be fully sequenced in 1994 [15]. In rats, neonatal BDV infection using that strain causes disturbances in learning, mood, and behavior reminiscent of those observed in human psychiatric diseases, making rodents a model system to study the consequences of persistent BDV infection [37]. In contrast, BDV natural strains like Hu-H1 were rare human wild-type isolates from PBMCs of psychiatric patients, recovered through co-cultivation with human OL cells. Passages of Hu-H1 from p11 on replicated to stable virus titers, and displayed different pathogenic phenotypes in animals [18] based on few meaningful mutations compared to lab strains [34]. Despite the sequence identity of RNA being derived from the original human PBMCs and the corresponding isolate (>passage p20), the authenticity of human strains has been questioned simply based on the high level of sequence conservation of BDV (>95%) in very short gene stretches instead of complete genome sequences [38]. Our recent *in vitro* studies rebutted this type of pure genetically based argumentation at the cellular level in that Hu-H1 inhibited cell proliferation and promoted apoptosis in OL cells, while Strain V did the opposite [24].

In this study, we have again focused on the comparison of these strains to further elucidate the molecular basis of BDV pathogenesis and strain differences. Through a GC–MS metabonomic approach coupled with PCA, PLS-DA, and OPLS-DA statistical analyses, this study revealed a set of differential metabolites that clearly distinguishes between the BDV Hu-H1, BDV Strain V, and non-infected controls. With regard to the fact that our study again provided strong evidence for complex biological strain differences, here shown through specific differential metabolic impact on neuronal cells, we for the first time introduce a systematic classification of natural BDV Hu-H1 and non-natural BDV Strain V. This classification approach followed the standardized taxonomic principles recently proposed for natural and laboratory strains of EBOV [21,22]. In light of the so far few natural strains of BDV of human and animal origin which are awaiting further biological characterization, this classification will provide a valuable template for better defining strains in future studies.

Below, we discuss the significant changes in metabolite levels in more detail. They were primarily observed in energy metabolites and amino acids. The neurons cultured were more than 90%, but need small numbers of astrocytes to grow properly. The identified metabolites are thus much more likely to be attributed to the overwhelming majority of neurons than to a few percent of astrocytes in the cultures.

### 3.1. Energy Metabolism

Both Hu-H1 and Strain V decreased citric acid, pyruvic acid, glycerol-3-phosphate, sorbitol, myo-inositol, and malic acid levels significantly but to different extents upon one of the two infections compared to control cells. The strains profoundly differed in that d-glucose, d-glucose-6-phosphate, d-fructose-6-phosphate, d-fructose, and d-lactose were significantly increased in the Hu-H1 group compared to controls, whereas d-glucose, d-glucose-6-phosphate, d-fructose-6-phosphate, and ribulose-5-phosphate were significantly decreased in the Strain V group compared to controls.

These findings clearly indicate divergent effects of the two BDV strains upon glucose metabolism in rat cortical neurons. Lower levels of glycolytic and TCA (tricarboxylic acid cycle) intermediates in conjunction with higher levels of upstream sugars in Hu-H1-infected neurons indicates an upstream equilibrium shift favoring gluconeogenesis. In contrast, in Strain V cells, lower levels of glycolytic and TCA intermediates in conjunction with lower levels of upstream sugars in Strain V-infected neurons indicate an equilibrium shift away from gluconeogenesis, glycolysis, and the TCA cycle (and perhaps toward ancillary pathways such as the pentose phosphate pathway, which supports nucleic acid synthesis). As mentioned above, our previous study found that Hu-H1 inhibits cellular proliferation and promotes apoptosis, while Strain V does the opposite using human oligodendrocytes [26]. The long-term host transition-derived adaptation of laboratory BDV Str. V may have manipulated these pathways to (i) produce ATP more efficiently through the aerobic pathway to support ATP-dependent nuclear protein import/export; (ii) promote *de novo* synthesis of membrane lipids for its lipid envelope; and (iii) promote *de novo* synthesis of nucleic acid monomers like those known for influenza virus infection [39]. Based on these combined findings, Strain V may be shunting carbon flux away from gluconeogenesis, glycolysis, and the TCA cycle to biosynthetic pathways to support cellular proliferation, whereas Hu-H1 does not.

In conclusion, metabolite changes vary significantly between natural and non-natural BDV strains. They vary as well between BDV and other viruses. In general, viral replication requires energy from the metabolic network of the host cell [40]. In terms of large DNA viruses like herpes viruses, metabolic flux studies have revealed that they are able to actively redirect energy metabolism in the host cell rather than passively relying on basal host cell metabolic activity [41]. Human cytomegalovirus (HCMV) and HSV-1 (Herpes Simplex Virus 1) infection significantly perturb glycolysis, TCA cycle, and pentose phosphate pathway intermediates in host cells. HCMV-infected cells have shown ribose-phosphate pyrophosphokinase up-regulation in infected cells. This is typical for rapidly replicating viruses suggesting the enyzme’s role in increasing the levels of *de novo* viral nucleic acid biosynthesis via the pentose phosphate pathway (PPP) [42]. In contrast, BDV Hu-H1 infection in our previous proteomic analysis was found to favor ribose 5-phosphate over its conversion into 5-phosphoribosyl-1-pyrophosphate (PRPP) that is the precursor to RNA synthesis [24]. This observation is consistent with a slow-replicating virus [4] which does not require high levels of *de novo* RNA synthesis. In an *in vivo* model, however, using the same human strain, some nucleotide metabolites involved in replication were elevated in three brain areas of post-natally infected rats at day 56 post infection [32]. While Strain V may be shunting carbon flux to biosynthetic pathways like the PPP to support cellular proliferation, thus behaving similarly to the influenza virus [39], Hu-H1 does not, thereby indicating a shift towards gluconeogenesis instead, as found in this study.

Altered energy metabolites and altered cell proliferation are interdependently linked. A good example of increased cell proliferation without virus infection is the development of cancer. Cancer cells are long known to reprogram the energy metabolism to meet their high demands in cell proliferation and biosynthesis. A recent review focused on the aforementioned pentose phosphate pathway (PPP) which has a neglected though important role in cancer growth [43]. The PPP is a major glucose catabolic pathway that links glucose metabolism to the biosynthesis of the nucleotide precursor ribose and to NADPH (nicotinamide adenine dinucleotide phosphate) production. To meet their biosynthetic demands, cancer cells are metabolically reprogrammed to direct glucose flux into the PPP. The same up-regulation of PPP is also seen in many virus infections as discussed above, promoting proliferation. Again, the divergence between the two BDV strains is striking in this respect.

### 3.2. Cholesterol and Fatty Acid Metabolism

Fatty acid profiling and cholesterol metabolism are of interest, particularly in cases of enveloped viruses. Lin *et al.* [44] found altered fatty acid and cholesterol signatures induced by influenza A virus infection. Interestingly, the cell types, either poorly differentiated AGS (gastric adenocarcinoma line) cells or a differentiated lung tumor cell line (A549), accounted for different metabolic shifts and replication efficiency. A large enveloped herpes virus, HCMV, notably increased the flux through the tricarboxylic acid cycle and its efflux to the fatty acid biosynthesis pathway. This turned out to be essential for viral replication and at the same time offered a target for antiviral therapy, identified through systems-level metabolic flux profiling [28]. In our study, at least the cholesterol metabolism has been addressed to some extent. A comparison of natural strain Hu-H1 with non-natural Strain V revealed a down-regulation of metabolites cholesterol, glycerol, desmosterol and lanosterol in Hu-H1, suggesting relatively less efficient production of enveloped viral particles. The same trend of down-regulated fatty acids and lipids as for Hu-H1 have been observed in the hippocampus of asymptomatic naturally infected horses [33]. In this study, desmosterol and margaric acid were down-regulated for either virus infection as compared to uninfected control cells, while eicosanoic acid and methylsuccinic acid showed up-regulation. Our data are not sufficient to estimate the impact of BDV in general and these differing strains in particular, on fatty acid and cholesterol biosynthesis pathways. Further studies are needed to elucidate whether natural BDV tends to lower the release of mature enveloped particles as compared to non-natural laboratory animal-adapted strains. Moreover, further studies on lipid metabolism and fatty acid biosynthesis will be of great interest, as differences to other enveloped viruses may be suggested from the preliminary results in this study.

### 3.3. Amino Acid Metabolism

**Figure 4:**
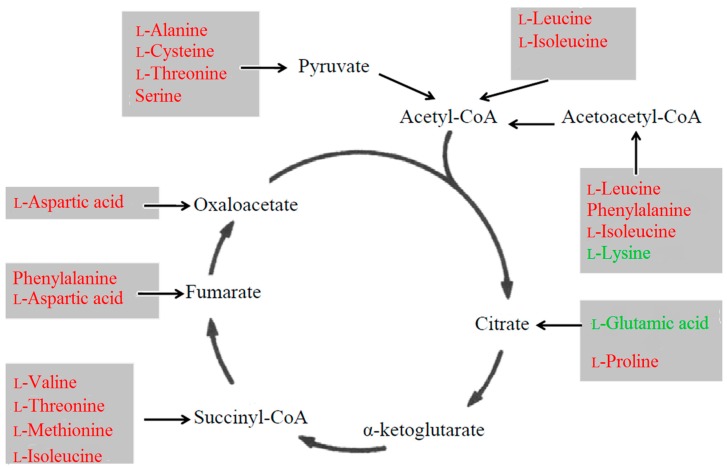
Relationship between perturbed amino acids and energy pathways. Red font indicates decreases in both the Hu-H1 and Strain V groups. Green font indicates decreases in the Strain V group alone.

All amino acid perturbations were significantly decreased both in Hu-H1 and Strain V compared to controls (Table 2 and Table 3). Only l-lysine and l-glutamic acid were decreased in Strain V-infected cells alone. Here, several perturbed amino acids are convertible with intermediates of glucose metabolism (Figure 4). For instance, the amino acid aspartate participates in gluconeogenesis through the malate-aspartate shuttle, which enables the introduction of aspartate and oxaloacetate in the TCA cycle. In addition, aspartic acid acts as a hydrogen acceptor in a chain of ATP synthase. Therefore, the decreases in these amino acids may reflect the aforementioned down-regulation in carbon flux through the TCA cycle.

Notably, we detected significant changes in four non-aromatic amino acids that function as CNS (central nervous system) neurotransmitters (γ-aminobutyric acid (GABA), l-aspartic acid, l-glutamic acid, and serine) as well as phenylalanine, which is an upstream precursor of two other CNS neurotransmitters, serotonin and dopamine [45]. This altered amino acid profile is noteworthy in light of previous studies linking other neurotropic viral infections with CNS neurotransmitter dysregulation [46]. GABA exists predominantly in the CNS as a primary inhibitory neurotransmitter [47], and previous studies indicate that the BDV P protein can bind directly and regulate GABA receptor-associated protein (GABARAP) [48].

In the mammalian CNS, glutamate and aspartate act as primary excitatory neurotransmitters at the *N*-methyl-d-aspartate (NMDA) receptor, while serine serves as a neuromodulator by co-activating the NMDA receptor [49]. Previous studies in a rat model showed that perturbations in these three neurotransmitters lead to NMDA receptor over-activation, inducing excitotoxicity and neurodegeneration in the hippocampal CA1 region [50,51]. Interestingly, the hippocampal distribution pattern of BDV’s proteins and RNA is restricted to the CA3 region and the dentate gyrus and is absent in the CA1 region [14]. Our findings are supported by a previous *in vivo* neonatal rat brain study that reported lower aspartate levels in BDV-infected subjects [52]. A decrease in glutamate levels was also consistent with our own previous findings in the hippocampi of asymptomatic horses [33]. Decreased intracerebral glutamate involves functional damage of the glutamatergic system. *In vivo*, not only neurons but also astrocytes could be severely impaired in their ability to uptake glutamate, thus leading to enhanced neuronal excitotoxicity. Inhibition of glutamate uptake by persistent BDV infection had been shown in feline primary cortical astrocytes infected with another non-natural laboratory animal-adapted strain (He/80) of equine origin, while glucose uptake and viability remained unchanged [53]. In conclusion, down-regulated amino acids, including those which function as neurotransmitters, appeared as a common pattern of both BDV strains in rat cortical neurons. In light of BDV’s affinity for glutamatergic neurons, the here-observed decrease in neuronal glutamate only found in non-natural Strain V is an interesting result, consistent with inhibited glutamate uptake *in vivo* through another non-natural strain [53]. Further studies are needed to elucidate whether decreased intracerebral glutamate is a pathogenic feature attributable to the species or the sub-species level of BDV.

## 4. Experimental Section

### 4.1. History and Proposed Taxonomy of Virus Strains

BDV Hu-H1 and BDV Strain V are distinct strains of completely different origin. The history of these strains are described in full detail in Table 1, following the recently published taxonomic principles for EBOV [21,22], the first application of which to BDV sub-species level was part of this work. Therefore, Table 1 and the entire history of the virus stocks used were put under the Section “Results”.

### 4.2. Primary Culture of Neurons and Viral Infections

Virus stocks of BDV Hu-H1 and Strain V in oligodendroglia cells as well as uninfected OL cells were kindly supplied by Hanns Ludwig (Free University of Berlin, Berlin, Germany) in 2010. As specified in Table 1, strain BDV Hu-H1-94 3660 stock virus (1994) in OL-221 was given at passage p75, and BDV Strain V stock virus (1998) in OL-221 was given at passage 113. Strain Hu-H1 was further propagated until passage p77 in OL-221 passage p112. Strain V was propagated until passage p115 in OL-221 passage p116, for each using strictly separated lamina air flow facilities of our laboratory at the Chongqing Medical University.

Cells obtained from the brains of Sprague-Dawley rats (post-natal day 1) were mixed and then distributed into poly-l-lysine-coated six-well plates (Sigma, Shanghai, China) at a density of 1.5 × 10^6^ cells/well in 35 wells (*n* = 11 infected with BDV Hu-H1 (Hu-H1 group), *n* = 12 infected with BDV Strain V (Strain V group) and *n* = 12 non-infected wells (CON group)). In brief, the brains were taken out and immersed in ice-cold Ca^2+/^Mg^2+^-free Hanks’s salt solution (HSS), pH 7.5. After removing the meninges, the cerebral cortical regions were dissected and dissociated by mild trypsinization (0.25% trypsin, Gibco, Shanghai, China) and DNase I (100 U/mL, Gibco) for 25 min. The cell fraction was suspended in Dulbecco’s Modified Eagle Medium (DMEM, Gibco) containing 10% fetal calf serum (Gibco), 10% horse serum (Gibco), 1% glutamine (HyClone, Shanghai, China), 0.1% penicillin (10 U/mL, HyClone), and 0.1% streptomycin (10 μg/mL, HyClone). Then, the cells were seeded on poly-l-lysine-coated six-well plates (Sigma, Shanghai, China) at a density described above. After four to six hours, the culture medium was replaced with a neurobasal medium (Gibco), including 2% B-27 (Gibco) for 12 h. Then, once this medium was removed, the cells were infected for 2 h with a multiplicity of infection (MOI) of 0.5 focus-forming units. BDV infection was performed by adding a cell-released virus (CRV) to the culture medium. CRV stocks were prepared as previously described [54]. The CON group was added to the cell lysis buffer without a virus as a parallel controlled experiment. Then, excess virus was removed by washing with 2 mL phosphate buffer saline (HyClone) before bathing the neurons again in a neurobasal medium. Thereafter, all cells were cultured in a humidified incubator (5% CO_2_, 37 °C) for 12 days. During this period, half of the medium was changed every three days.

### 4.3. Immunofluorescence

Standard immunofluorescence was performed as described previously [55]. Briefly, both BDV-infected and control neurons were incubated on six-well plates for 30 min at room temperature with 4% paraformaldehyde followed by permeabilization for five minutes in 0.25% Triton X-100. After permeabilization, both neurons were rinsed with phosphate buffer saline (PBS) three times and blocked with 5% bovine serum albumin (BSA) for 30 min. Incubation for one hour at room temperature with the neuron-specific markers MAP-2 and anti-BDV-specific p40 antigen primary monoclonal antibody was performed [56]. After several washes in PBS, one hour incubation at room temperature with secondary antibodies was performed. After extensive washing, the neurons were counterstained with DAPI. After three PBS washes, immunofluorescence was detected using an inverted fluorescence microscope (Nikon, Tokyo, Japan).

### 4.4. Metabolite Extraction

On day 12 post-infection, the medium was aspirated out, and the cells were rinsed twice with 2 mL of ice-cold PBS. The cells were digested by trypsin for 2 min at 37 °C. After decanting trypsin, 2 mL of 4 °C PBS was added to the well. Then the cell suspension was transferred to 1.5 mL centrifuge tubes and centrifuged at 3000 rpm for 10 min. The cell pellets were stored at −80 °C. For GC–MS analysis, each frozen cell sample was added to 1 mL of chromatographic grade methanol and 10 μL of Dulcitol-13C6 as an internal standard. After vortexing for 30 s and storing at 4 °C overnight, the mixture was sonicated for 15 min and subsequently centrifuged at 14,000 rpm for 15 min at 4 °C. Then, a 400 μL volume of supernatant was evaporated to dryness under a stream of nitrogen gas. The dried residue was dissolved in 30 μL methoxamine hydrochloride (20 mg/mL pyridine) and incubated at 37 °C for 90 min with continuous shaking. Subsequently, the solution was derivatized with 30 mL BSTFA (Bis trimethylsilyl trifluoroacetamide) + 1% TMCS (trimethyl chlorosilane) at 70 °C for 60 min and then placed at room temperature for 30 min before GC–MS analysis.

### 4.5. Gas Chromatography–Mass Spectrometry (GC–MS) Analysis

Each 1 μL of the derived sample was injected into an Agilent 7890A GC system (Agilent Technologies Inc., Santa Clara, CA, USA). An HP-5 MS fused silica capillary column (30 m × 0.25 mm × 0.25 mm, (Agilent Technologies Inc.), within which a helium carrier gas flowed at a rate of 0.6 mL/min, was applied for metabolite separation. The temperatures of the injector, the EI iron source, and quadrupole rods were set at 280, 230 and 150 °C, respectively. The column temperature was initially maintained at 80 °C for 2 min and then raised to 320 °C by 10 °C/min where it was held for 6 min. The column effluent was introduced into the ion source of an Agilent 5975 mass selective detector (Agilent Technologies Inc.). Masses were acquired from *m*/*z* 50 to 600. To avoid the influence induced by instrument signal fluctuations, a random order of continuous sample analysis was adopted.

### 4.6. Metabonomic Data Analysis

GC–MS metabolite profiles were processed after conversion into a netCDF file format using TagFinder [57]. This process enabled deconvolution, alignment, and data reduction to produce a list of mass and retention time pairs with the corresponding intensities for all detected peaks from each data file in the data set. The resulting table was exported into Microsoft Excel where normalization was performed, and the resulting three-dimensional data set—including peak index (RT-*m*/*z* pair), sample names (observations), and normalized peak area percentages—was imported to SIMCA-P 11.0 (Umetrics, Umea, Sweden) for statistical analysis.

Principal components analysis (PCA) was applied to the unit variance (UV)-scaled spectral data to reveal intrinsic cell-related patterns. Partial least squares discriminant analysis (PLS-DA) with UV-scaled spectral data was also performed in order to improve classification and to provide pair-wise comparison between groups. Then, OPLS-DA [58] was applied to decrease the effects of non-relevant metabolite variability and classify BDV infection samples from control samples. OPLS-DA produced two key parameters: R2Y (the cumulative model variation in Y) and Q2 (the cumulative predicted variation). As these parameters approached 1.0, a stable model with predictive reliability was indicated. The discriminating metabolites were identified based on a statistically significant threshold of variable influence on projection (VIP) values from the OPLS-DA model (VIP > 1) and two-tailed Student’s *t*-test (*p* < 0.05). Fold change was calculated as the logarithm of the average mass response (area) ratio between groups.

## 5. Conclusions

In conclusion, employing a GC–MS-based metabonomic approach, this is the first study to show that BDV infection with natural Hu-H1 and non-natural Strain V differentially alters metabolic pathways of rat cortical neurons *in vitro*. Comparative metabonomic profiling revealed statistically significant perturbations in key energy and amino acid metabolites that were divergent between the two BDV strains. Upon differing origin and adaptation levels, the two BDV strains may differentially manipulate the host neuron’s metabolic network to support differing viral replication and proliferation strategies. These remarkably different effects of a natural human and a horse-derived cross-species-adapted laboratory strain of BDV on neuronal cell metabolism found in our study also provide new avenues for future exploration of the pathogenic mechanism of BDV infection *in vivo*. With regard to the importance of using defined viruses, as emphasized by our findings, a systematic taxonomical nomenclature to classify these viruses is introduced for the first time, providing a valuable template for defining strains in future studies.
